# Suspension Cell Culture of *Polyscias fruticosa* (L.) Harms in Bubble-Type Bioreactors—Growth Characteristics, Triterpene Glycosides Accumulation and Biological Activity

**DOI:** 10.3390/plants12203641

**Published:** 2023-10-22

**Authors:** Maria V. Titova, Dmitry V. Kochkin, Elena S. Sukhanova, Elena N. Gorshkova, Tatiana M. Tyurina, Igor M. Ivanov, Maria K. Lunkova, Elena V. Tsvetkova, Anastasia Orlova, Elena V. Popova, Alexander M. Nosov

**Affiliations:** 1 K.A. Timiryazev Institute of Plant Physiology, Russian Academy of Sciences, 127276 Moscow, Russiaorlova@ifr.moscow (A.O.); popova@ifr.moscow (E.V.P.); al_nosov@mail.ru (A.M.N.); 2 Biology Faculty, M.V. Lomonosov Moscow State University, 119234 Moscow, Russia; 3 Department of Biochemistry, Saint Petersburg State University, 199034 Saint Petersburg, Russia; e.v.tsvetkova@spbu.ru; 4 Department of General Pathology and Pathological Physiology, Institute of Experimental Medicine, 197022 Saint Petersburg, Russia

**Keywords:** *Ming aralia*, PFS, ladyginoside A, antimicrobial activity, antioxidant activity, cell aggregation, cell farming, plant cell biotechnology

## Abstract

*Polyscias fruticosa* (L.) Harms, or Ming aralia, is a medicinal plant of the Araliaceae family, which is highly valued for its antitoxic, anti-inflammatory, analgesic, antibacterial, anti-asthmatic, adaptogenic, and other properties. The plant can be potentially used to treat diabetes and its complications, ischemic brain damage, and Parkinson’s disease. Triterpene glycosides of the oleanane type, such as 3-*O*-[β-*D*-glucopyranosyl-(1→4)-β-*D*-glucuronopyranosyl] oleanolic acid 28-*O*-β-*D*-glucopyranosyl ester (PFS), ladyginoside A, and polysciosides A-H, are mainly responsible for biological activities of this species. In this study, cultivation of the cell suspension of *P. fruticosa* in 20 L bubble-type bioreactors was attempted as a sustainable method for cell biomass production of this valuable species and an alternative to overexploitation of wild plant resources. Cell suspension cultivated in bioreactors under a semi-continuous regime demonstrated satisfactory growth with a specific growth rate of 0.11 day^−1^, productivity of 0.32 g (L · day)^−1,^ and an economic coefficient of 0.16 but slightly lower maximum biomass accumulation (~6.8 g L^−1^) compared to flask culture (~8.2 g L^−1^). Triterpene glycosides PFS (0.91 mg gDW^−1^) and ladyginoside A (0.77 mg gDW^−1^) were detected in bioreactor-produced cell biomass in higher concentrations compared to cells grown in flasks (0.50 and 0.22 mg gDW^−1^, respectively). In antibacterial tests, the minimum inhibitory concentrations (MICs) of cell biomass extracts against the most common pathogens *Staphylococcus aureus*, methicillin-resistant strain MRSA, *Pseudomonas aeruginosa*, and *Escherichia coli* varied within 250–2000 µg mL^−1^ which was higher compared to extracts of greenhouse plant leaves (MIC = 4000 µg mL^−1^). Cell biomass extracts also exhibited antioxidant activity, as confirmed by DPPH and TEAC assays. Our results suggest that bioreactor cultivation of *P. fruticosa* suspension cell culture may be a perspective method for the sustainable biomass production of this species.

## 1. Introduction

*Polyscias fruticosa* (L.) Harms (Syns = *Panax fruticosa* = *Nothopanax fruticosum* = *Parsley panax* = *Ming aralia*) belonging to family Araliaceae, is a medicinal plant widespread in the Polynesian islands of the Pacific region and Southeast Asia (India, Malaysia, Indonesia, [1]. The name *Polyscias* is composed of two Greek words: ‘poly’ meaning many, and ‘skia’ meaning shade, indicating the dense foliage typical for this genus [1]. The plant grows relatively slowly with stem reaching one or two meters tall; the roots resemble parsley in smell and taste [2]. *P. fruticosa* is sensitive to stresses, particularly temperature stress, and has an optimal temperature range of 19–29 °C [3].

Roots and leaves of *P. fruticosa* contain triterpene glycosides, alkaloids, vitamins, amino acids, cyanogenic glycosides, polyacetylenes, sterols, tannins, essential oils, essential micro- and macroelements, and sugars [1,4,5,6]. Triterpene glycosides are represented mainly by the oleanane-type compounds, such as 3-*O*-[β-*D*-glucopyranosyl-(1→4)-β-*D*-glucuronopyranosyl] oleanolic acid 28-*O*-β-*D*-glucopyranosyl ester (PFS), ladyginoside A (LadA), and polysciosides A-H [1,7,8]. According to Do et al. [9], in 2019, methyl-glucuronate glycosides polyscioside J and polyscioside K and chikusetsusaponin IVa were isolated and identified in the leaves of *P. fruticosa* for the first time.

*P. fruticosa* is actively used in traditional medicine due to a wide range of pharmacological effects and safety: according to Koffuor et al. [10], the No-Observable-Adverse Effect-level (NOAEL) of the ethanolic leaf extract is below 1000 mg kg^−1^. Over the past 20 years, studies have demonstrated antitoxic, anti-inflammatory, analgesic, and molluscicidal properties of *P. fruticosa* extracts [10,11,12,13] and their antibacterial activity against Gram-positive and Gram-negative bacteria [13,14]. The high potential of using *P. fruticosa* for treating diabetes and its complications has been reported in the in vivo models [11,15,16,17]. *P. fruticosa* leaf extract also exhibited anti-asthmatic and antihistamine activities increasing protection against histamine-induced bronchospasm and reducing recovery time [10,18]. Roots are used to treat neuralgia and rheumatic pain, to improve brain function, and as diuretic and anti-dysentery agent [19]. In old male rats, root extract stimulated sexual activity and increased fertility, and significantly increased memory function and lifespan [20,21,22]. *P. fruticosa* leaf and root extracts stimulated the immune system to produce antibodies or interferons, thus enhancing the body’s defense against diseases, and exhibited adaptogenic activity due to modulation of the body’s endocrine system to counteract stress [1,14]. Several studies reported the neuroprotective effect and therapeutic potential of *P. fruticosa* against ischemic brain damage and Parkinson’s disease [23,24].

In addition to medicinal purposes, *P. fruticosa* is widely used as a spice and as a fresh vegetation for salads, as well as in tonic drinks and tea [25,26]. Several studies investigated changes in the phytochemical characteristics of *P. fruticosa* during drying and storage to select optimal conditions for its processing and preservation [25,27,28].

Despite the high potential value of *P. fruticosa* for functional foods and pharmacology, information on in vitro cultivation of this species is scarce. Several protocols have been published on in vitro micropropagation using shoot tips, and stem and leaf explants [2,3,29]. Vinh et al. [30] worked on the optimization of culture medium to enhance the growth and biosynthetic characteristics of *P. fruticosa* callus. Kim et al. [31] investigated the effects of carbon sources and plant growth regulators on growth and oleanolic acid accumulation in *P. fruticosa* cell suspension cultures. Phuong et al. [32] examined the effects of two auxins, 2,4-dichlorophenoxyacetic acid (2,4-D) and α-naphthaleneacetic acid (NAA), on biomass accumulation, rhizogenesis, and somatic embryogenesis of *P. fruticosa* suspension-cultured cells. Hau et al. [33] observed the accumulation of oleanolic acid (40.1 µg g^−1^) and saponins (396.2 µg g^−1^) in proliferated *P. fruticosa* hairy roots. Adventitious root induction was also reported for this species [34].

Production of suspension cell biomass in bioreactors could potentially provide a sustainable, renewable source of plant material compared to quantitatively limited and chemically variable wild plants [35,36,37,38,39]. However, to our knowledge, there are no publications on the cultivation of *P. fruticosa* cells in bioreactors.

In the previous studies, we reported the development of callus and suspension cell cultures of *P. fruticosa* in flasks followed by biochemical analysis of their triterpene glycosides (TG) content [40,41,42,43,44]. The present study aimed to establish a suspension cell culture of *P. fruticosa* in 20 L laboratory bubble-type bioreactors to explore the feasibility of upscaling the cell biomass production and its potential use as a novel food supplement and a source of TG. The content of two main TG in *P. fruticosa* cell biomass, as well as the antioxidant and antimicrobial activities of the cell biomass extracts, were evaluated for quality control.

## 2. Results

### 2.1. Suspension Cell Culture Growth in Flasks

**Figure 1:**
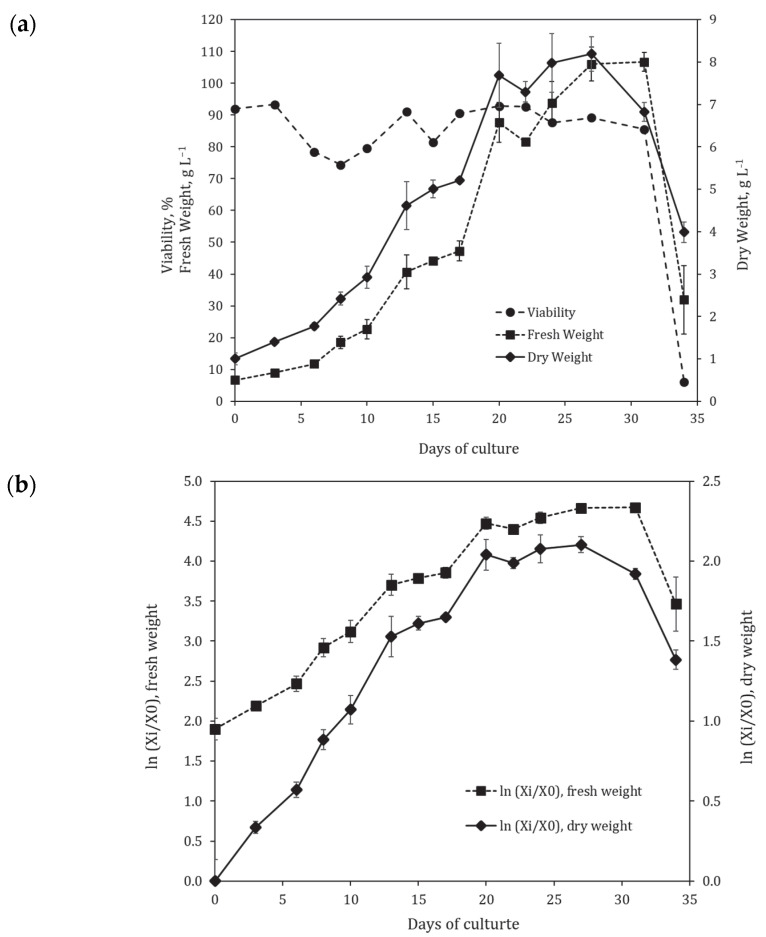
Representative growth curves of the suspension cell culture of *Polyscias fruticosa* during cultivation in 250 mL flasks: (**a**) fresh and dry weights and cell viability plotted in normal coordinates; (**b**) fresh and dry weights plotted in semi-logarithmic coordinates.

The suspension cell culture of *Polyscias fruticosa* has been maintained as a flask culture since 2005, and its growth characteristics were monitored periodically [40,41,42,43,44]. The growth dynamics of the cell culture in 250 mL flasks was recorded before [43] and in parallel with bioreactor cultivation. The representative growth curves in normal and semi-logarithmic coordinates are shown in Figure 1a,b, respectively. Particularly for this part of the research, the cultivation was extended to over 30 days to record the complete growth cycle, including the degradation phase.

At the initial inoculum density near 1.1 g L^−1^, the lag phase (~2 days) and acceleration phase (~4 days) were observed. The duration of the exponential growth phase was 7–9 days followed by the 7-day stationary phase, and then the degradation phase. Cell viability during the cultivation cycle varied within the 75–95% range, followed by a sharp dropdown after 31 days during the degradation phase.

The main growth parameters for the suspension cell culture in flasks are presented in Table 1. The culture grew well, with average maximum biomass accumulation of 8.2 g L^−1^, specific growth rate of 0.21 day^−1^, and productivity of 0.45 g (L · day)^−1^ calculated based on dry weight (DW).

**Figure 2:**
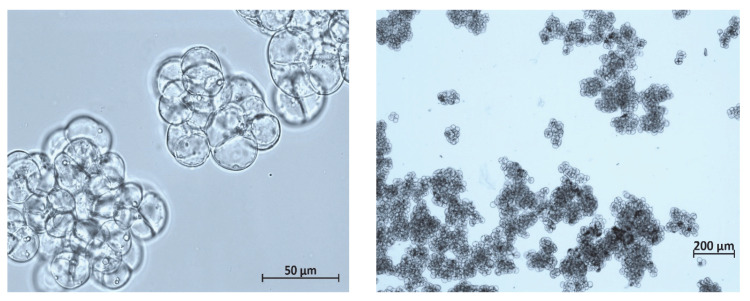
Representative photographs of cells and cell aggregates of *Polyscias fruticosa* suspension cell culture grown in flasks at different magnifications.

The cytological analysis revealed that cells in the suspension were, on average, relatively small (20–35 µm in diameter) and round-shaped. Typical microphotographs of cells in the exponential growth phase (day 10) are presented in Figure 2. Cell aggregates were predominantly round with uneven edges. The aggregation level (percentage of aggregates consisting of a certain number of cells) estimated at the early exponential phase (day 10) and beginning of the stationary phase (day 20) is shown in Table 2. The culture was relatively small-aggregated: about 70% of the aggregates in the suspension consisted of less than 50 cells, and the aggregates were less than 1 mm in diameter. The number of viable single cells and small aggregates (up to 5 cells) was insignificant (Table 2).

Based on growth and the cytological data, the suspension cell culture was considered potentially suitable for the cultivation in bioreactors.

### 2.2. Suspension Cell Culture Growth in 20 L Bioreactor under a Semi-Continuous Cultivation Regime

**Figure 3:**
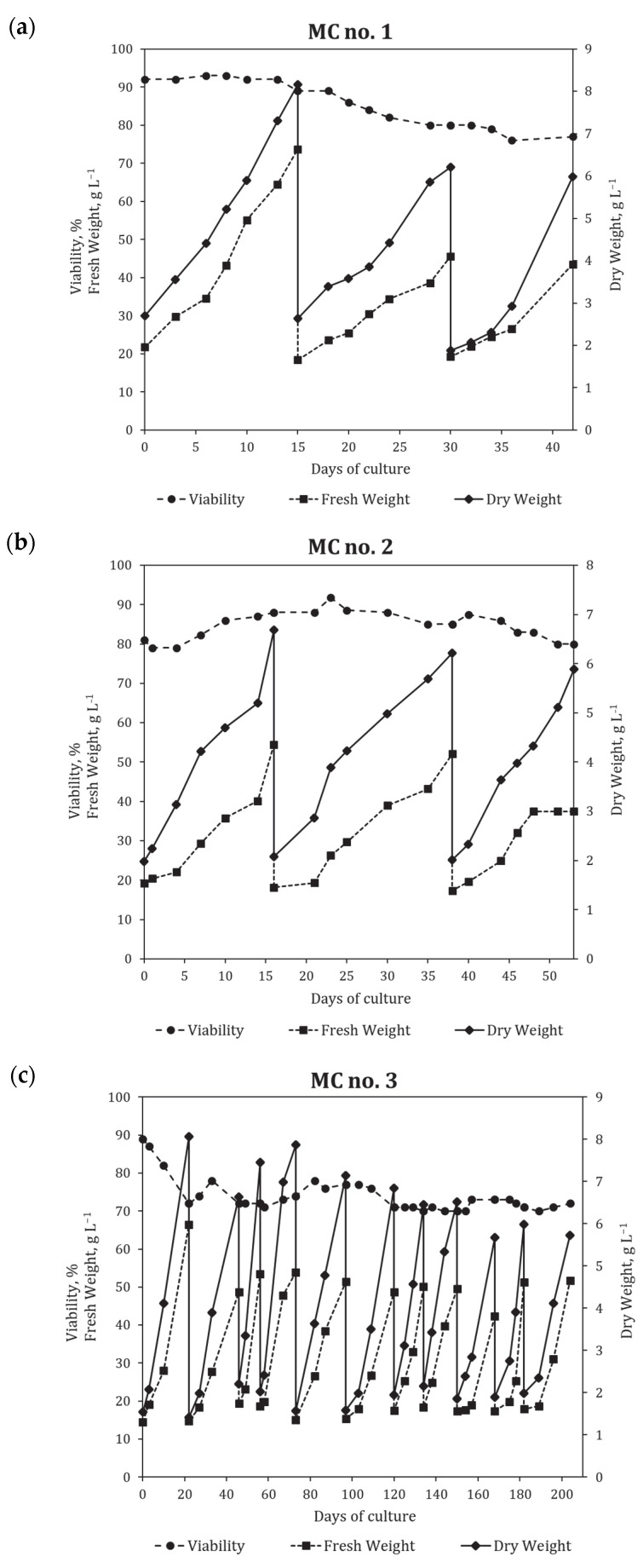
Growth curves of *Polyscias fruticosa* suspension cell culture during cultivation in 20 L bioreactor in a semi-continuous cultivation regime: (**a**) Multi-cycle (MC) no. 1; (**b**) MC no. 2; (**c**) MC no. 3.

To establish the bioreactor cultivation for cell suspension of *P. fruticosa* in bubble-type 20 L glass bioreactors, three sequential multi-cycle (MC) runs were performed under a semi-continuous regime. The first two MC runs consisted of three subculture cycles, and the third MC run had 11 subculture cycles (Figure 3). In each subculture cycle, a portion of the suspension was harvested at the end of the exponential—beginning of the stationary growth phase, and the remaining suspension was diluted by adding fresh medium to reach the suspension concentration around 1.5–2.0 gDW L^−1^ that allowed further growth without a lag phase. The total duration of multicycles 1, 2, and 3 was 42, 53, and 204 days, respectively. The growth curves and main growth characteristics of the cell culture during MC 1, 2, and 3 are shown in Figure 3 and Table 1 and Table 2.

Growth parameters of the cell suspension in bioreactors changed compared to flasks (Table 1). An increase in inoculum density to ~2 g L^−1^ allowed to omit the lag phase and thus shorten the cultivation time until reaching a maximum biomass production point from 22 to 16–17 days on average (Table 1) while keeping a nearly similar maximum biomass concentration level (*X_max_* = 6.7 g L^−1^ in bioreactors vs. 8.2^−1^ in flasks). However, the specific growth rate of the culture in bioreactors decreased almost twice compared to flasks. Some reductions in productivity (by 22%) and economic coefficient (by 41%) were also recorded. Despite the overall good cell viability (above 70%) during cultivation in bioreactors, a steady decrease in maximum fresh weight (FW) and DW concentrations was observed within each MC run, and this tendency persisted through all three MC runs performed in this study (Figure 3). However, growth parameters were not significantly different between three independent MC bioreactor runs.

Compared to flask culture, a minor shift towards higher aggregation was observed for the cell suspension in bioreactors, as the percentage of aggregates with over 50 cells increased from 27.6 to 39.3% (Table 2). However, this change had no negative impact on the technological aspects of the cultivation process.

### 2.3. Analysis of the Two Major Secondary Metabolites in the Suspension Cell Culture of Polyscias fruticosa during Cultivation in Flasks and Bioreactors

**Figure 4:**
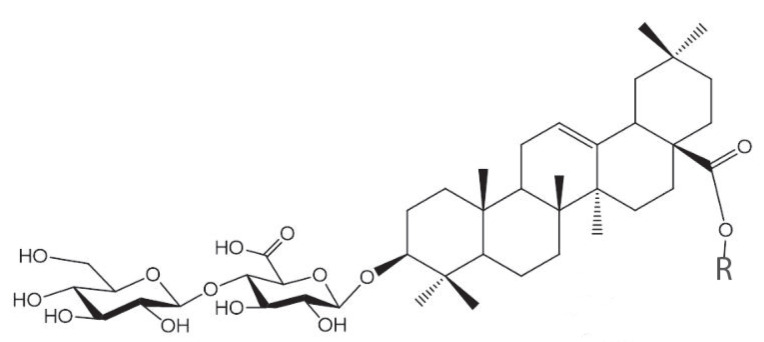
Chemical structure of triterpene glycosides found in cell biomass of *Polyscias fruticosa*: Ladyginoside A: R = H; PFS: R = β-D-glucopyranoside.

Triterpene glycosides ladyginoside A and PFS (28-*O*-β-*D*-glucopyranosyl ester of oleanolic acid 3-*O*-β-*D*-glucopyranosyl-(1→4)-β-*D*-glucuronopyranoside) in dried cell biomass of *P. fruticosa* were identified as described earlier [40,41,42] using UPLC-ESI-MS based on the interpretation of corresponding mass-spectra, comparison of chromatographic behavior, and mass-spectra with standard samples previously isolated from the leaves of *Polyscias filicifolia* [40,41,42] and the literature data [45,46]. Structural formulas of the two compounds are presented in Figure 4. The results of qualitative and quantitative analysis of triterpene glycosides (negative ion mode) in cell biomass are presented in Table 3 and Table 4. Ladyginoside A and PFS were detected in cell biomass during cultivation in flasks and bioreactors (Table 4). Interestingly, the production of both compounds in bioreactors was significantly higher compared to flasks (*p* = 0.01 for PFS and *p* = 0.0002 for LadA). In flasks, PFS accumulation in cell biomass was higher at day 20 (end of exponential phase) compared to day 27 (end of stationary phase–beginning of degradation phase). At the same time, the content of LadA did not change significantly between these growth phases. The content of LadA remained relatively stable within each multi-cycle bioreactor run, ranging from 0.65 to 0.75 mg gDW^−1^ depending on a subcultivation cycle. The content of PFS varied in a broader range (0.70–1.17 mg gDW^−1^) between subcultivations within one MC run. However, the average content of both triterpene glycosides was similar between the three MC rungs in bioreactors (Table 4), indicating relatively good reproducibility of biosynthesis of TG in the bioreactor cultivation system.

### 2.4. Antioxidant and Antimicrobial Activities of the Extracts from Cell Biomass of P. fruticosa Grown in Bioreactors

For rapid assessment of the biological activity of the cell biomass extracts, their antimicrobial and antioxidant activities were determined and compared to those of leaves of the greenhouse plants.

The minimum inhibitory concentrations (MICs) of cell biomass extracts against the most common pathogens, *Staphylococcus aureus*, including methicillin-resistant strain MRSA, *Pseudomonas aeruginosa*, and *Escherichia coli*, are given in Table 5. Cell biomass was most effective against *E. coli*, followed by *S. aureus* and *P. aeruginosa*, and less effective against MRSA. However, for all pathogens tested, the antimicrobial activity of cell biomass extracts exceeded those exhibited by extracts from greenhouse plant leaves.

The antiradical activity of cell biomass and greenhouse plant leaf extracts was determined by two spectrophotometric tests, DPPH and TEAC, based on the ability of plant material samples to bind stable radicals. The *P. fruticosa* leaf extract showed higher antiradical activity in both tests, and the obtained absolute effective concentrations causing a 50% effect (EC_50_) of extracts from leaves were lower than the EC_50_ of extracts from cell culture biomass (Table 6).

## 3. Discussion

Our results demonstrated that the suspension cell culture of *P. fruticosa* grew well in flasks (Table 1). The maximum dry weight accumulation in flasks achieved in our study (8.2 g L^−1^ at the inoculum density 1.1 g L^−1^) was similar to what was previously recorded for the same cell strain [40,44] and to those reported for the suspension cell cultures of *P. fruticosa* by Kim et al. [31] (0.33–0.45 g per flask or 6.5–9.0 g L^−1^ depending on the media composition at inoculum density 3.3–3.4 g L^−1^). The cell culture showed a lag phase when subcultured with inoculum density about 1.0 g L^−1^ [44]. The growth parameters of the *P. fruticosa* suspension cell culture were good enough to consider its bioreactor cultivation. For example, the economic coefficient of 0.27 suggests that almost one third of sucrose supplied to culture medium is utilized for building cell structures and compartments.

The size of cell aggregates in the suspension culture is essential for bioreactor cultivation. Large cell aggregates can lead to an inhomogeneous distribution of nutrients, oxygen, light, and other environmental factors between the cells, thus reducing the suspension growth. In addition, large cell aggregates create technological problems in bioreactor operation, such as excessive sedimentation and adhesion of cells to bioreactor walls and hose plugging [47,48]. In the present study, the suspension cell culture of *P. fruticosa* had no large aggregates (over 1 mm in diameter) throughout the growth cycle in flasks, which was a positive factor for further upscaling to bioreactors (Table 3). During bioreactor cultivation, the ratios of cell aggregate groups changed towards forming larger aggregates. However, such change had no impact on the cultivation process due to the small size of the cells.

For bioreactor cultivation, the bubble type of bioreactors and the semi-continuous cultivation regime were selected based on a review of the literature data on the classification of bioreactors and their technological parameters as well as on the results of our experience with bioreactor cultivation of the cell suspensions of other plant species [35,36,49,50,51]. The aeration regime for bioreactors was selected experimentally. The airflow rate varied depending on the cell suspension growth stage, taking into account the following requirements: (i) the concentration of dissolved oxygen (pO_2_) should remain above 15%; (ii) there should be no stagnant (“dead”) zones in the bioreactor, no cell sedimentation, and no intensive foaming [52,53]. Dissolved oxygen concentrations between 15 to 20% air saturation are usually recommended for plant cell cultivations [54].

Inoculum density is one of the main parameters for optimizing bioreactor cultivation for plant suspension cell cultures. Several studies revealed that variations in the inoculum size can lead to changes in culture growth kinetics and production of secondary metabolites and cell biomass, and this effect is specific for different cell cultures, even within the same plant species [55,56,57,58]. Increasing the inoculum density to a certain level leads to a reduction in or complete disappearance of the lag phase, an increment in the specific growth rate, earlier entrance to the stationary growth phase, and earlier synthesis of secondary metabolites. On the contrary, reducing the inoculum below a certain critical value, specific for each cell culture, may lead to a significant suppression and, in some cases, a complete arrestment of the culture growth. In the present study, higher inoculum density was used for bioreactor cultivation of *P. fruticosa* cell suspension compared to flask cultures (2.07 vs. 1.10 gDW L^−1^). This allowed omitting the lag phase and reducing the time required to reach the maximum biomass accumulation point, thus resulting in 25–30% shorter subculture cycles compared to flask culture. However, it also led to reduction in growth parameters (productivity, specific growth rate, and economic coefficient). A similar tendency was observed for flask cultures where inoculum size above 2.5 gDW L^−1^ led to a significant slowdown in cell culture growth, a decrease in viability and maximum biomass accumulation (Supplementary Figure S1), and medium darkening during 2–3 subcultivation cycles. Therefore, in the present study, inoculum size was controlled at ~1.8–2.4 gDW L^−1^, and the fresh medium was fed into bioreactors at a cell suspension concentration of 6.5–8.5 gDW L^−1^. Factors limiting the growth of cell cultures during cultivation with high inoculum density may include rapid depletion of nutrients in the culture medium, accumulation of toxic byproducts, tissue metabolites, or dead cell residues.

The maximum biomass accumulation and the average specific growth rate decreased in bubble-type bioreactors compared to flask culture (Table 2). It is likely that the growth limitations may be associated with higher stress levels in the bubble-type bioreactors in comparison with flasks [37,54,59]. However, the type of bioreactors used in the present study was selected based on our previous successful experience with suspension cell cultures of a number of medicinal species, such as *Dioscorea deltoidea*, *Panax japonicus*, *Stephania glabra*, *Taxus wallichiana,* and a relative species *Polyscias filicifolia* [35,60,61,62]. These cultures retained their main characteristics during cultivation in bioreactors of this type. Unlike cell cultures mentioned, a cell suspension of *P. fruticosa* requires further optimization of the bioreactor conditions and a more comprehensive investigation of cell suspension physiology. At the same time, the growth and biomass accumulation parameters were stable and repetitive between multicycles performed during three years, which makes the future of bioreactor cultivation of this cell strain quite promising.

It is essential that the cell culture grown in bioreactors accumulate biologically active compounds and maintain active biosynthesis of the desired secondary metabolites. Qualitative and quantitative analysis of secondary metabolites confirmed the presence of TG of the oleanolic acid group in cell biomass samples from both flasks and bioreactors. These compounds were PFS and LadA, identified earlier in this suspension cell culture [40,42,44] (Table 3). During the bioreactor cultivation, the suspension cell culture of *P. fruticosa* retained active synthesis of these metabolites at a sufficiently high level, exceeding those recorded during cultivation in flasks. With increasing duration of bioreactor cultivation (MC 3 in Table 4), a relatively stable synthesis was observed for LadA. In contrast, PFS content showed fluctuations within a 0.20–0.40 mg gDW^−1^ range between the subculture cycles. These results are in accordance with other studies [1,7,8,16] reporting oleanolic acid saponins as major components of *P. fruticosa*. PFS and LadA were found in the leaves and roots of *P. fruticosa* plants [7,8,9]. Moreover, the PFS saponin is quantitatively the primary compound isolated from leaves and roots of *P. fruticosa* plants. Therefore, PFS can be potentially used as a marker for quality control of *P. fruticosa*-based products, particularly cell biomass [8]. It is worth noting that the amount of PFS in the cell culture in our study was comparable to its content in leaves (1.30 mg gDW^−1^) and roots (0.57 mg gDW^−1^) of *P. fruticosa* plants [8].

It is acknowledged that the oleanolic acid glycosides are primarily responsible for the biological activity of *P. fruticosa* [10,11,12,13,14], including antibacterial effects. In particular, *P. fruticosa* leaf extract exhibited antibacterial activity on Gram-positive Cocci *Staphylococcus aureus* (including MRSA), *Bacillus subtilis*, and *E. coli* [13,14]. It is considered that oleanolic acid saponins can increase and/or disrupt the permeability of the bacterial cell membrane, which leads to damage and death of the bacterial cells [13]. In our work, the antibacterial activity of extracts of cell suspension biomass from bioreactors was investigated in vitro by broth microdilution assay using cultures of the four most common pathogens. The highest antibacterial activity for cell biomass extract was observed against the Gram-negative strain *Escherichia coli* (MIC 250 μg mL^−1^). The lowest activity was revealed against the Gram-positive strain MRSA ATCC 33591 (MIC 2000 μg mL^−1^). Importantly, the antimicrobial activity of the extracts from cell biomass exceeded those of extracts from plant leaves (Table 5).

Several publications reported the in vitro and in vivo antioxidant activities of crude extracts of *P. fruticosa* leaves and roots [24,27,63,64]. There are different methodologies for evaluating the antioxidant activity of both synthetic and natural compounds. In our study, we performed DPPH and ABTS assays as rapid and low-cost methods for antioxidant activity screening of cell biomass extracts. These methods are frequently used for the evaluation of the antioxidative potential of plant extracts, including most of the published studies on the antioxidant activities of *P. fruticosa* [24,27,28,63,65]. In our study, cell biomass extracts demonstrated moderate antioxidant activities that were slightly lower than the activity measured for extracts from leaves of the greenhouse plants (Table 6). These differences can be attributed to the activity of both TG found in cell biomass and polyphenolic compounds (not analyzed in the present study). For example, Tran et al. [27] demonstrated strong correlations between the antioxidant activity of the *P. fruticosa* extract and the content of polyphenols and triterpenoid saponins.

## 4. Materials and Methods

### 4.1. Cell Suspension Cultivation in Flasks and Bioreactors

**Figure 5:**
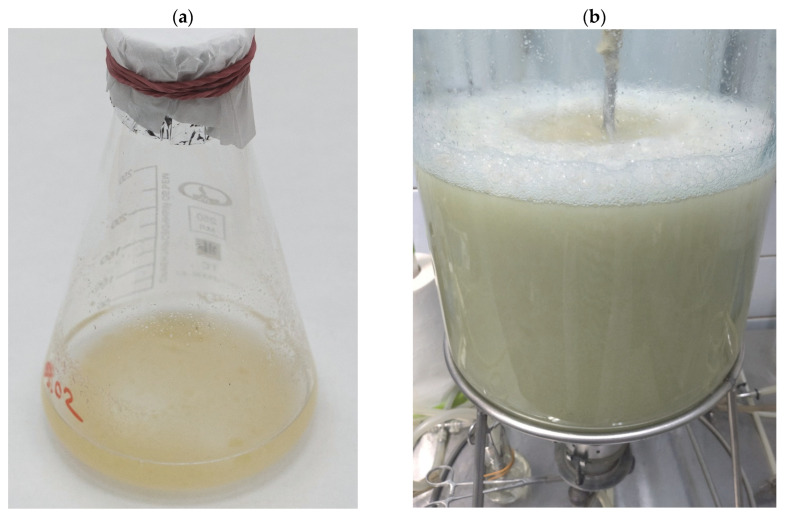
Suspension cell culture of *Polyscias fruticosa*: (**a**) in 250 mL flasks; (**b**) in 20 L bubble-type bioreactors.

*Polyscias fruticosa* (L.) Harms suspension cell culture, strain 6a, was provided by the All-Russian Collection of Plant Cell Cultures at the Institute of Plant Physiology of the Russian Academy of Sciences (Moscow, Russia). The strain was induced from a leaf of a greenhouse plant, as described earlier [43]. Stock cell culture was maintained in 250 mL Erlenmeyer flasks filled with 30 mL of modified Murashige and Skoog liquid medium with 30 g L^−1^ sucrose and plant growth regulators 2,4-D (2 mg L^−1^) and BA (1 mg L^−1^) [43,66] (Figure 5, Table 7). The initial density (inoculum) size was set at 0.9–1.2 gDW L^−1^. Cultures were grown on an orbital shaker (95–100 rpm) at 26–27 °C and 70–75% relative air humidity in darkness. Subcultures were performed every 14 days. In the experiments on growth curve estimation, the culture period was extended to 34 days to capture the degradation phase.

Bubble-type 20 L glass bioreactors (Institute of Plant Physiology of RAS, Moscow, Russia) with 15 L working volume and air supply through a sparger were used to cultivate cell suspension (Table 7; Figure 5). The choice of bioreactor type was based on previous studies demonstrating that barbotage bioreactors produced minimum mechanical damage to plant cell suspensions [35,60,67]. The scheme of the bioreactor is given in Supplementary Figure S2. Cultivation was performed using a semi-continuous regime at 26 ± 0.5 °C in darkness. To maintain the semi-continuous cultivation, a portion of cell suspension was regularly harvested from bioreactors using a special sterile hosepipe at the beginning of the stationary growth phase of a subcultivation cycle, which was measured as cell suspension concentration reaching 6.5–8.5 gDW L^−1^. Simultaneously, the sterile fresh nutrient medium was added to bioreactors until cell suspension was diluted to 1.5–2.0 gDW L^−1^, and the cultivation process was continued. This process was repeated when the cell suspension reached the beginning of the stationary phase of the next subcultivation cycle. Each bioreactor cultivation under a semi-continuous regime thus consisted of multiple subcultivation cycles and is therefore designated in the text as a “multi-cycle (MC) run.” Three independent MC runs of bioreactors were performed in this study. The first two MC runs consisted of three subculture cycles, and the third MC run had 11 subculture cycles (Figure 3). The total duration of multicycles 1, 2, and 3 was 42, 53, and 204 days, respectively.

The dissolved oxygen (pO_2_) concentration was maintained at 10–40% of saturation volume without intense foaming. Air was supplied to bioreactors at a rate varying from 0.1 to 1.0 v vpm^−1^ depending on the growth phase of the cell culture. The minimum air flow rate was set at the beginning of subculture cycles to reduce the adverse effects of intensive suspension mixing while avoiding cell sedimentation. During the exponential growth phase, the air supply rate was increased to the maximum possible that did not cause cell destruction, as confirmed by microscopic observations.

### 4.2. Assessment of Growth and Physiological Characteristics of the Cell Suspension Culture

During cultivation in flasks and bioreactors, FW and DW of cell biomass, aggregation level, and viability were recorded every 2–3 days during the cultivation cycle [60]. For each time point, three flasks or three samples from bioreactors were taken (*n* = 3).

To estimate the FW, 10–15 mL of the cell suspension was collected on paper filters in a Büchner funnel, and the culture medium was removed under vacuum. Cells were washed three times with distilled water under vacuum, and FW of the biomass sample was recorded. To estimate DW, cell biomass sample was dried at 40 °C for 48 h to a constant weight. The biomass samples for chemical analysis and biological activity assessment (see below) were prepared following the same procedure.

Cell viability was calculated under microscope after staining with 0.025% Evans blue (modified from [68]) as the percentage of cell aggregates composed of colorless (living) cells. Aggregation was defined as the ratio of different types of aggregates expressed in percentages. To increase contrast, cells were stained with 0.1% phenosafranin, and the number of aggregates of different types was counted under a light microscope. The diameter of large aggregates was measured under a binocular microscope. The measurements were performed in three biological and two analytical repetitions (a fixed volume of cell suspension was taken twice from each of the three flasks or bioreactor samples, and each time at least 100 aggregates were measured).

In addition, the following growth parameters were calculated based on DW according to [35,69]:

Specific growth rate, indicating the rate of dry weight increase per day:

*µ* = maximum value of *µ_i_* = ln(*X_i_*/*X_i−_*_1_)/(*t*_i_
*−*
*t_i_*_−1_), [day ^−1^]

where *X_i_* and *X_i−_*_1_ are, respectively, dry cell biomass concentrations (g L^−1^) at time points *t*_i_ and *t_i_*_−1_;

Doubling time, indicating the time requiring for doubling dry cell weight at constant *µ* (exponential growth phase):

*τ* = ln2/*µ*, [day]

Productivity on dry biomass, indicating the amount of biomass that can be harvested from one liter of the suspension per day:

*P* = maximum value of *P_i_* = (*X_i_* − *X*_0_)/(*t_i_ − t_0_*) [g (L day)^−1^]

where *X_i_* and *X_0_* are, respectively, dry cell biomass concentrations (g L^−1^) at time points *t_i_* and at the time of inoculation *t_0_*

Economic coefficient, indicating the efficiency of substrate (sucrose) utilization for cell growth:

*Y* = (*X_max_* − *X_0_*)/*S_0_*,

where: *X*_0_ and *X_max_* are, respectively, initial and maximum dry cell biomass concentrations (g L^−1^); *S_0_*—initial sucrose concentration in the medium (g L^−1^).

### 4.3. Extract Preparation and High-Performance Liquid Chromatography-Electrospray Ionization–Mass Spectrometry (UPLC-ESI-MS) Analysis of Triterpene Glycosides in Cell Biomass

Dried cell biomass was powdered, and extraction and purification by solid-phase extraction were performed according to previously published procedures [70]. The evaporated extracts were dissolved in 2 mL of 70% (*v*/*v*) aqueous methanol and filtered through a nylon filter “Acrodisc” with 0.2 µm pores (Pall Corporation, NY, USA).

The analysis of triterpene glycosides was performed using ACQUITY UPLC H-Class PLUS chromatograph (Waters, Milford, MA, USA) coupled with electrospray ionization and a hybrid time-of-flight mass spectrometry detector Xevo G2-XS TOF (Waters, Milford, MA, USA). Samples (0.05 µL) were separated on the ACQUITY UPLC BEH C18 column (50 × 2.1 mm, 1.7 µm; Waters, Drinagh, County Wexford, Ireland) at 40 °C with mobile phase flow rate 0.4 mL min^−1^. The composition of the mobile phase was: 0.1% (*v*/*v*) solution of formic acid in water (phase A) and 0.1% (*v*/*v*) solution of formic acid in 99.9% (*v*/*v*) acetonitrile (phase B). Phases were prepared using deionized water (Simplicity UV, Millipore, Molsheim, France) and acetonitrile of “LC-MS” grade (Panreac, Barcelona, Spain). Analytes were separated using a gradient (B, % by volume): 0–1 min—5→15%, 1–5 min—15→30%, 5–11 min—30→38%, 11–15 min—38→65%, 15–15.5 min—65→95%.

Analysis was performed in a negative ion detection mode in the *m/z* range of 100–1900. The ionization source temperature was set to 150 °C, desolvation temperature 650 °C, capillary voltage 3.0 kV, sample injection cone voltage 30 V; desolvation gas (nitrogen) flow rate 1101 L h^−1^. Data analysis was performed using MassLynx software 4.2 (Waters, Milford, MA, USA).

Triterpene glycosides were identified using cell culture grown in flasks as described earlier [41]. Structural identification of ladyginoside A and PFS (28-*O*-β-*D*-glucopyranosyl ester of oleanolic acid 3-*O*-β-*D*-glucopyranosyl-(1→4)-β-*D*-glucuronopyranoside) was carried out by comparing chromatographic and mass spectrometric characteristics of the found compounds with standard samples of these glycosides, previously isolated from the leaves of *Polyscias filicifolia* and the structure of which was unambiguously confirmed by 1D and 2D NMR and high-resolution mass spectrometry [41]. The concentrations of individual compounds in cell biomass grown in flasks and bioreactors were determined from the calibration curves constructed using the external standard of ginsenoside R_0_ (Sigma-Aldrich, MA, USA). Standard deviations for retention time and chromatographic peak squares were below 1% and 15%, respectively. In a working concentration range (0.02–50.00 µg mL^−1^), linear models approximated the calibration curve with correlation coefficients R^2^ > 0.995.

### 4.4. Test Systems for Rapid Assessment of the Biological Activity of Cell Biomass Extracts: Antioxidant and Antimicrobial Tests

#### 4.4.1. Preparation of Extracts from Plant Leaves and Cell Culture Biomass for Antioxidant and Antimicrobial Activity Tests

Antioxidant and antimicrobial activities of cell biomass harvested from a 20 L bioreactor were compared to those of greenhouse *P. fruticosa* plant leaves. Mature leaves were randomly collected from two pot plants (five per plant) growing in a greenhouse of the Main Botanic Garden (Moscow, Russia) and air-dried at 40 °C under the same conditions as cell biomass. Dried cell and leaf biomass samples were ground to a fine powder in a mortar. Samples weighing 2 g were extracted in a 20-fold (1:20) volume of 100% and 80% aqueous methanol in an ultrasonic bath (Sapfir, Russia) for 15 min each. The total extract was filtered, evaporated under vacuum at 40 °C, freeze-dried, and stored at 5 °C. For antimicrobial activity tests, samples were re-dissolved in a 10% dimethyl sulphoxide (DMSO) solution. The resulting extracts (20 mg mL^−1^) were diluted by distilled water to a final concentration of 4 mg mL^−1^ and used in the tests. For the antioxidant activity assay, dry extracts prepared as described above were re-dissolved in 4% DMSO to obtain a stock solution of 100 mg mL^−1^. These solutions were further diluted to a range of concentrations (from 7 to 12 mg mL^−1^ for the leaf extract and 5 to 20 mg mL^−1^ for the cell culture extract) and used to determine the EC_50_ values in DPPH and TEAC assays.

#### 4.4.2. Determination of Antioxidant (Antiradical) Activities Using DPPH and TEAC Assays

The antioxidant (antiradical) activity of the suspension cell culture and leaf extracts were determined by 2,2-diphenyl-1-picrylhydrazyl (DPPH) free radical scavenging and Trolox equivalent antioxidant capacity (TEAC) assays. The analyses were performed according to Masci et al. [71], with minor modifications as follows. All chemicals were analytical grade and purchased from Sigma. Absorbance was measured using a spectrophotometer Genesys 20 (Thermo Scientific, USA).

##### DPPH Assay

An amount of 10 µL of extract solutions diluted as described in 4.4.1. was added to 1 mL of 40 µmol L^−1^ methanolic solution of stable nitrogen-centered free radical DPPH^∙^. The absorbance was monitored spectrophotometrically at 517 nm after one hour of incubation at room temperature in the dark. The capacity for scavenging the DPPH-radical was estimated from the difference between the absorbance measured with and without the addition of the extract.

##### TEAC Assay

A 7 mmol L^−1^ solution of the 2,2′-azinobis(3-ethylbenzothiazoline-6-sulfonic acid) diammonium salt (ABTS) was prepared by dissolving ABTS in water. Then, ABTS was oxidized to a radical cation (ABTS^+∙^) by 2.45 mmol L^−1^ potassium persulfate during 16 h incubation at room temperature in the dark. The radical cation reagent (ABTS^+∙^) was diluted with ethanol to obtain an absorbance of 0.70 ± 0.02 at 734 nm. To initiate the reaction, 10 µL of extract solutions diluted in 4% DMSO, as described above, were added to 1 mL of the ABTS^+∙^ solution. The mixture was incubated for six minutes in the dark and at room temperature, and absorbance was measured at 734 nm. The antioxidant capacity of the samples was expressed in Trolox equivalents.

##### Estimation of the Effective Concentration (EC_50_)

Antiradical activity curves were plotted referring to sample concentrations on the *X* axis and their relative radical scavenging capacity on the *Y* axis. Effective concentration (EC_50_) was defined as a sample concentration that gives a 50% percentage effect. The EC_50_ values were determined using a linear regression algorithm in GraphPad Prism version 8.0.1. The experiments were performed in triplicate, and the actual EC_50_ was estimated by calculating the average value from three different EC_50_ extrapolations with correlation coefficients R^2^ > 0.80.

#### 4.4.3. Determination of Antibacterial Activities

The antibacterial activity analysis was modified from Orlova et al. [72]. Bacterial strains used were *Escherichia coli* ATCC 25922, *Pseudomonas aeruginosa* ATCC 27853, and *Staphylococcus aureus* ATCC 25923, MRSA ATCC 33591. Strain MRSA ATCC 33591 was provided by Prof. R. Lehrer (University of California Los Angeles, CA, USA); *Escherichia coli* ATCC 25922, *Pseudomonas aeruginosa* ATCC 27853, *Staphylococcus aureus* ATCC 25923 were provided by the Department of Molecular Microbiology, IEM. Bacterial strains were cultured under aerobic conditions according to the approved standard protocols. The minimum inhibitory concentrations (MIC) of the cell and leaf extracts were determined by the broth microdilution method, as recommended by the Clinical Laboratory Standards Institute, USA [73]. The microorganisms were transferred from an agar plate culture to 2.1% (*w/v*) Mueller–Hinton broth (MHB, HiMedia, Mumbai, India) and incubated on an orbital shaker (150 rpm) at 37 °C for 2–6 h. After adjusting the turbidity to 0.5 McFarland (1.5 × 10^8^ CFU mL^−1^), suspensions were diluted in sterile 2.1% (*w/v*) MHB until achieving a final bacterial concentration of 1.0 × 10^6^ CFU mL^−1^.

The cell suspension and leaf extracts were serially two-fold diluted (starting with the initial concentration 4 mg mL^−1^) with sterile 2.1% (*w/v*) MHB. Then, 25 µL aliquots were placed in the wells of a 96-well sterile U-shaped plate (GreinerBio-one, Austria). Afterward, bacterial suspension (25 µL) was added to each well. The microtiter plates were incubated in a thermostat at 37 °C for 18 h. MICs were defined as the lowest extract concentrations that inhibited the visual growth of microorganisms. The experiments were performed in triplicate, and the final results were calculated as the medians based on the data from three independent experiments, each accompanied by the complete set of the controls of bacterial growth and viability, and medium sterility.

### 4.5. Statistical Analysis

Data on suspension cell growth are presented as the mean values and standard deviations recorded for the triplicates (three flasks or three fixed-size samples of cell suspension collected from bioreactors) for each data point. For viability assessment, a minimum of 250 cell aggregates were examined in each of the three replicates for every experimental condition. Growth parameters and triterpene glycosides’ content during bioreactor cultivation were determined as the average of several subcultivation cycles within each multi-cycle bioreactor run. Standard deviations that constitute less than 10% of mean values are not displayed in the graphs. Antioxidant and antimicrobial tests were performed in triplicate.

The data analysis for this paper was generated using SAS software. Copyright© 2023 SAS Institute Inc. SAS and all other SAS Institute Inc. product or service names are registered trademarks or trademarks of SAS Institute Inc., Cary, NC, USA. Statistical significance of differences was estimated using the one-way ANOVA test followed by the Duncan Multiple Range test at *p* < 0.05 for growth parameters and Dunnett’s test at *p* < 0.05 for triterpene glycoside content.

## 5. Conclusions

This is the first study on bioreactor cultivation of *P. fruticosa* suspension cell culture. The results demonstrated that suspension cell culture of this species can be grown in laboratory (20 L) bubble-type bioreactors under a semi-continuous regime for at least 200 days. Cell culture retained satisfactory growth and biosynthetic abilities and accumulated oleanolic acid triterpene glycosides (ladyginoside A and PFS). Cell biomass extracts exhibited antibacterial activity against four cultures of the most common pathogens. In antibacterial tests, the minimum inhibitory concentrations (MICs) of cell biomass extracts against the most common pathogens, *Staphylococcus aureus*, methicillin-resistant strain MRSA, *Pseudomonas aeruginosa*, and *Escherichia coli* varied within 250–2000 µg mL^−1^ which was higher than in leaves of greenhouse plants (MICs = 4000 µg mL^−1^). Cell biomass extracts also exhibited antioxidant activity, as confirmed by DPPH and TEAC assays. The results of this study will be useful for further optimization of bioreactor cultivation for this species. Our data also suggest that cell biomass of *P. fruticosa* produced in bioreactors has excellent potential to become a sustainable source of the vegetative raw material of this species for different purposes, for example, for functional foods, cosmetics, and natural health products and therefore, help reduce overexploitation of wild plants in their native habitats.

## Figures and Tables

**Table 1:** Growth parameters of the suspension cell culture of *Polyscias fruticosa* during cultivation in 250 mL flasks and 20 L bioreactors (calculated based on dry weight).

Parameter	Cultivation System				
	250 mL Flasks	20 L Bioreactors *			
		MC 1	MC 2	MC 3	Average of MC 1–3
*X_0_*, g L^−1^	1.10 ± 0.45	2.40 ± 0.37	1.97 ± 0.10	1.83 ± 0.27	2.07 ± 0.30
*∆t_max_*, days	22.5 ± 3.5 ^a^	15.0 ± 2.5 ^b^	16.5 ± 4.0 ^ab^	18.5 ± 4.5 ^ab^	16.7 ± 2.0
*X_max_*, g L^−1^	8.19 ± 0.81 ^a^	7.31 ± 1.43 ^a^	6.31 ± 0.34 ^a^	6.76 ± 0.81 ^a^	6.79 ± 0.50
*μ*, day^−1^	0.21 ± 0.03 ^a^	0.10 ± 0.02 ^b^	0.13 ± 0.03 ^b^	0.11 ± 0.02 ^b^	0.11 ± 0.03
*τ*, day	3.3 ± 0.4 ^b^	6.9 ± 1.2 ^a^	5.3 ± 1.6 ^ab^	6.3 ± 1.3 ^a^	6.3 ± 1.4
*Y*	0.27 ± 0.05 ^a^	0.16 ± 0.05 ^b^	0.14 ± 0.01 ^b^	0.16 ± 0.03 ^b^	0.16 ± 0.01
*P_max_*, g (L · day)^−1^	0.45 ± 0.12 ^a^	0.33 ± 0.06 ^ab^	0.30 ± 0.05 ^b^	0.35 ± 0.15 ^ab^	0.32 ± 0.13

MC—multi-cycle (continuous cultivation in bioreactors under semi-continuous mode consisting of three (MC 1 and 2) or 11 (MC 3) subcultivation cycles), *X_0_—*inoculum density, *∆tmax*—time from the beginning of the subculture cycle to achieving maximum biomass accumulation, *Xmax*—maximum dry cell weight, *μ*—the maximum value of the specific growth rate calculated for the analyzed growing cycle, *τ*—doubling time, *Y—*economic coefficient, *Pmax*—productivity. * For each MC, parameters were calculated as an average of all subcultivation cycles within the respective MC. Mean values in rows followed by the same letter are not significantly different at *p* < 0.05, according to Duncan’s multiple range test.

**Table 2:** Aggregation level of *Polyscias fruticosa* suspension cell culture during cultivation in 250 mL flasks and 20 L bioreactor estimated at the early exponential phase (day 10) and the beginning of the stationary phase (day 20, only for flasks). Aggregation level is presented as the percentage of aggregates consisting of a certain number of cells out of the total number of cell aggregates counted.

Day of the Growth Cycle	Number of Cells in the Aggregate					
	1–5	6–10	11–20	21–30	31–50	>50
	% of Aggregates Consisted of a Given Number of Cells					
	**250 mL flasks**					
Day 10	4.3 ± 2.1 ^a^	12.0 ± 2.0 ^a^	17.0 ± 1.0 ^a^	15.0 ± 6.2 ^a^	24.3 ± 3.2 ^a^	27.6 ± 5.5 ^b^
Day 20	2.3 ± 0.6 ^a^	5.7 ± 1.5 ^b^	13.0 ± 1.7 ^ab^	17.7 ± 8.4 ^a^	29.0 ± 4.3 ^a^	33.0 ± 6.9 ^ab^
	**20 L bioreactors**					
Day 10	4.0 ± 1.7 ^a^	7.7 ± 1.5 ^b^	10.0 ± 3.6 ^b^	15.4 ± 7.4 ^a^	25.3 ± 7.6 ^a^	39.3 ± 2.9 ^a^

Mean values in columns followed by the same letter are not significantly different at *p* < 0.05, according to Duncan’s multiple range test.

**Table 3:** Results of UPLC–ESI–MS analysis (negative ion mode) of extracts from cell biomass of *Polyscias fruticosa* suspension cell culture grown in flasks.

t_R_, min *	[M-H]^−^, m/z **	Identification Results
7.906	955.6	PFS ***
13.440	793.6	Ladyginoside A (LadA)

* Retention time on the chromatographic column (min); ** The *m/z* values for the ions detected in the mass spectra; *** 28-*O*-β-*D*-glucopyranosyl ester of oleanolic acid 3-*O*-β-*D*-glucopyranosyl-(1→4)-β-*D*-glucuronopyranoside.

**Table 4:** Content of PFS and ladyginoside A, as determined using UPLC–ESI–MS, in cell biomass samples of *Polyscias fruticosa* suspension cell culture grown in flasks and 20 L bioreactors.

Cultivation System	Variant	Triterpene Glycoside Content, mg gDW^−1^	
		PFS	Ladyginoside A
Flasks	Day 20	0.50 ± 0.05 ^bc^	0.22 ± 0.01 ^b^
	Day 27	0.34 ± 0.12 ^c^	0.20 ± 0.01 ^b^
Bioreactors *	MC no. 1	0.78 ± 0.01 ^a^	0.66 ± 0.01 ^a^
	MC no. 2	1.03 ± 0.37 ^a^	0.75 ± 0.08 ^a^
	MC no. 3	0.92 ± 0.15 ^ab^	0.79 ± 0.21 ^a^
	Average of MC 1-3	0.91 ± 0.12	0.73 ± 0.07

* For each MC, values are means of triterpene glycoside content measured during individual subcultivation cycles at the time points of maximum biomass accumulation; PFS—28-*O*-β-*D*-glucopyranosyl ester of oleanolic acid 3-*O*-β-*D*-glucopyranosyl-(1→4)-β-*D*-glucuronopyranoside; MC—multi-cycle. Mean values in columns followed by the same letter are not significantly different at *p* < 0.05, according to Duncan’s multiple range test.

**Table 5:** The minimum inhibitory concentration (MICs) of extracts from *Polyscias fruticosa* cell biomass grown in bioreactors and greenhouse plant leaves.

Extracts	MICs, µg mL^−1^			
	*Escherichia coli* ATCC 25922	*Staphylococcus aureus* ATCC 25923	MRSA ATCC 33591	*Pseudomonas aeruginosa* ATCC 27853
Cell biomass	250	500	2000	500
Plant leaves	4000	4000	4000	4000

MRSA—methicillin-resistant *Staphylococcus aureus.*

**Table 6:** Antioxidant activities of the extracts from *P. fruticosa* cell biomass and greenhouse plant leaves expressed as EC_50_ values (absolute effective concentrations causing a 50% effect), based on DPPH and TEAC assays.

Extracts	Assay *	
	DPPH EC_50_, mg mL^−1^	TEAC EC_50_, mg mL^−1^
Cell biomass	17.85 ± 0.44 ^a^	11.10 ± 0.98 ^a^
Plant leaves	10.69 ± 0.09 ^b^	8.81 ± 0.13 ^a^
Positive control	0.150 ± 0.002	0.036 ± 0.001

* DPPH—2,2-diphenyl-1-picrylhydrazyl free radical scavenging assay; TEAC—Trolox equivalent antioxidant capacity. Ascorbic acid and Trolox were positive controls for DPPH and TEAC assays, respectively. In columns, values followed by the same letter are not significantly different at *p* < 0.05 according to Student’s unpaired *t*-test with Welch correction.

**Table 7:** Major characteristics of cultivation systems used for *Polyscias fruticosa* cell suspension (based on [60]).

Cultivation System	250 mL Flasks on an Orbital Shaker	20 L Bioreactors (Bubble-Type)
Mode of operation	Batch	Semi-continuous
Working volume	35 mL medium	15 L
Aeration device	No	Sparger * n_h_ = 1 d_h_ = 6.0 mm

* Air supply 0.5 to 1.0 v vpm^−1^; n_h_—number of holes in the sparger; d_h_—diameter of the hole in the sparger.

## Data Availability

Raw data are available from authors upon request.

## Supplementary Materials

The following supporting information can be downloaded at: https://www.mdpi.com/article/10.3390/plants12203641/s1, Figure S1: Growth curves (dry weight) of the suspension cell culture of *Polyscias fruticosa* in 250 mL flasks plotted in semi-logarithmic coordinates. *X*_0—_initial dry cell biomass concentration (inoculum size); *X*—dry cell biomass concentration at time of sampling. Figure S2: Scheme of a bubble-type bioreactor used for the cultivation of *Polyscias fruticosa* cell suspension. 1—exhaust air, 2—sterile compressed air input (sparger diameter 6 mm), 3—sterile nutrient medium input, 4—sterile compressed air input for clearing sample collection pipeline, 5—pipeline for collecting cell suspension samples. A, B, C—air sterilization filters; D—container with sterile liquid medium. Bioreactor dimensions: larger diameter—225 mm, height—480 mm, mouth outer/inner diameter—60/46 mm.
