# Supplementary material for: Suspension Cell Culture of Polyscias fruticosa (L.) Harms in Bubble-Type Bioreactors—Growth Characteristics, Triterpene Glycosides Accumulation and Biological Activity
Source: Plants (Basel). 2023 Oct 22;12(20):3641. doi: 10.3390/plants12203641 (PMC10610180; doi:10.3390/plants12203641)
Supplement: Supplementary file 1 [file plants-12-03641-s001.zip › plants-2633309-supplementary.pdf]

Suspension Cell Culture of Polyscias fruticosa (L.) Harms in Bubble-Type Bioreactors—Growth Characteristics, Triterpene Glycosides Accumulation and Biological Activity

Maria V. Titova, Dmitry V. Kochkin, Elena S. Sukhanova, Elena N. Gorshkova, Tatiana M. Tyurina, Igor M. Ivanov, Maria K. Lunkova, Elena V. Tsvetkova, Anastasia Orlova, Elena V. Popova, Alexander M. Nosov

Supplementary materials

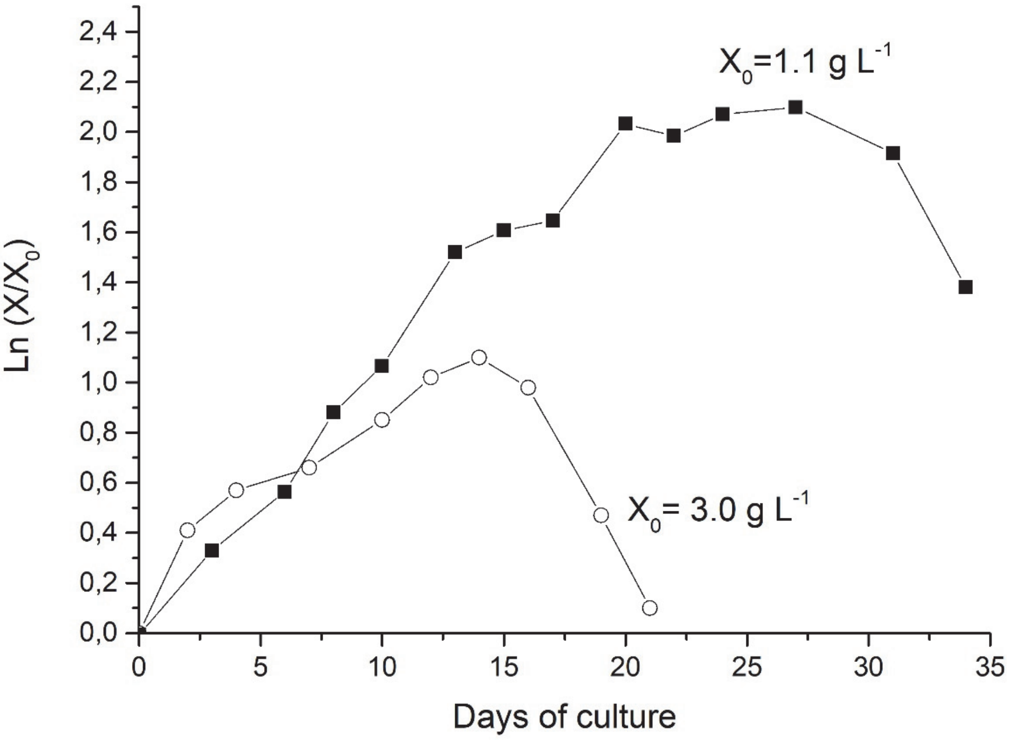

Figure S1. Growth curves (dry weight) of the suspension cell culture of *Polyscias fruticosa* in 250 ml flasks plotted in semi-logarithmic coordinates. X_0 – initial dry cell biomass concentration (inoculum size); X – dry cell biomass concentration at time of sampling.

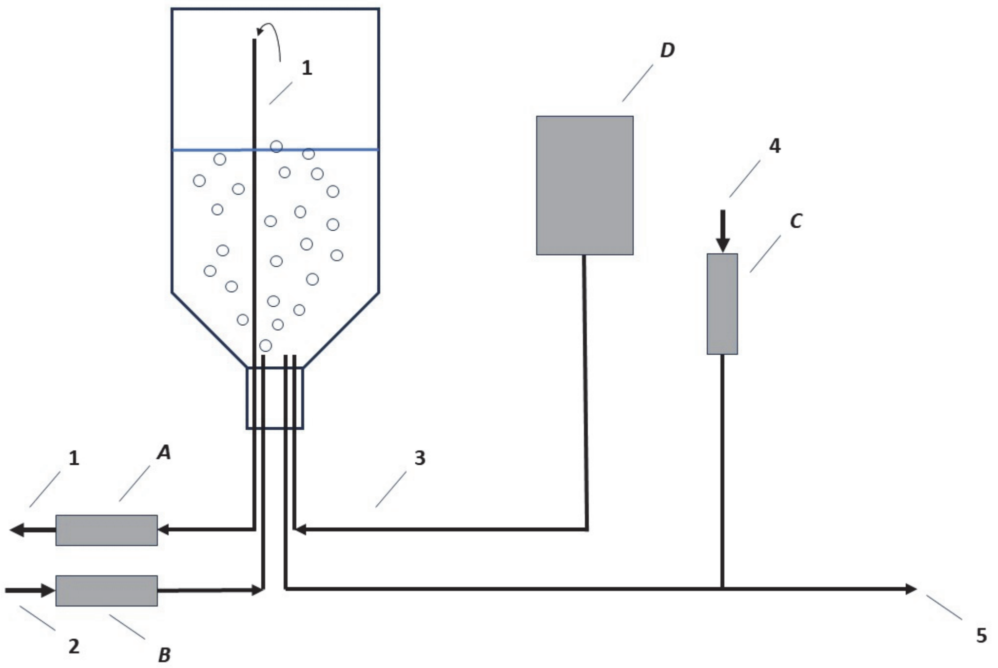

Figure S2. Principle scheme of a bubble-type bioreactor used for the cultivation of *Polyscias fruticosa* cell suspension. 1 – exhaust air, 2 – sterile compressed air input (sparger diameter 6 mm), 3 – sterile nutrient medium input, 4 – sterile compressed air input for clearing sample collection pipeline, 5 – pipeline for collecting cell suspension samples. A, B, C – air sterilization filters; D – container with sterile liquid medium. Bioreactor dimensions: larger diameter – 225 mm, height – 480 mm, mouth outer/inner diameter – 60/46 mm.
